# Influence of Pristine and Photoaging Polystyrene Microspheres on Sperm Quality and DNA Integrity of the Sand Dollars *Scaphechinus mirabilis*

**DOI:** 10.3390/jox15060176

**Published:** 2025-10-23

**Authors:** Andrey Alexandrovich Mazur, Sergey Petrovich Kukla, Victor Pavlovich Chelomin, Valentina Vladimirovna Slobodskova, Nadezhda Vladimirovna Dovzhenko

**Affiliations:** V.I. Il’ichev Pacific Oceanological Institute, Far Eastern Branch, Russian Academy of Sciences, Vladivostok 690041, Russia; mazur.aa@poi.dvo.ru (A.A.M.);

**Keywords:** gametes, genotoxicity, microplastics, polystyrene, sand dollar

## Abstract

Plastic pollution represents a significant emerging environmental problem. Micro-sized particles of synthetic polymers—microplastics (MPs)—have been identified in all parts of marine ecosystems. In the marine environment, organisms are exposed to MPs, which undergo a constant process of physicochemical and biological degradation. Utilization of UV irradiation as the optimal exposure factor in the simulation of fundamental natural conditions is a widely accepted approach. This enables the study of the harmful effects of such particles when interacting with aquatic organisms. This study aimed to investigate the effect of pristine and photoaging primary polystyrene microspheres (µPS) at three concentrations on the viability and DNA integrity of the sperm of the sand dollars *Scaphechinus mirabilis*. The results of the investigation demonstrated that IR spectroscopy revealed structural changes in polystyrene, confirming the oxidative degradation of the polymer under UV irradiation. The study demonstrated that artificially aged µPS exhibited a more pronounced effect than pristine particles, as evidenced by reduced sperm viability and increased DNA damage. Thus, the resazurin test showed that after exposure to UV-irradiated µPS, sperm viability decreased to 83–85% at concentrations of 10 and 100 particles and to 70% at a concentration of 1000. In addition, the Comet assay showed that the particles increased the percentage of DNA in the tail from 20% to 30% in a dose-dependent manner. The findings substantiate and augment the existing body of experimental data of the toxicity of aged plastic fragments, thereby underscoring the need for further study into the toxicity of aged MPs on marine invertebrates.

## 1. Introduction

Plastic items are now used worldwide for countless fields, including construction, medicine, and transport, due to their low production costs. Since the 1950s, global plastic production has grown exponentially. In 2022, approximately 400 million tons of plastic products were produced [1]. It is an irrefutable fact that most of the plastic products that are produced inevitably end up in the environment, particularly in the world’s oceans, where huge accumulations of countless items are formed. It is estimated that between 5 and 12 million tons of plastic waste of varying sizes end up in the oceans every year [2].

Plastic pollution is a major ecological problem. Plastic waste that remains in natural conditions for a long time changes its original properties under the influence of physicochemical and biological factors. This is a growing scientific concern. Such pieces inevitably become stiff and brittle and break down into progressively smaller particles, ranging in size from a few millimeters (no more than 5) to a few microns, commonly referred to as microplastics (MPs) [3]. Micro-sized polystyrene particles (µPS) have been incorporated into a wide range of products due to their high tensile strength, swelling capacity, stable chemical properties, regenerative properties, large surface area, and economic feasibility [4,5]. Penetrating into aquatic organisms, µPS causes serious toxic damage. It has been proven that µPS can block the secretion of digestive enzymes, leading to disorders of the immune system and reproductive functions. It can also cause changes in eating behavior and, as a result, slow down growth and development [6,7].

In recent years, a growing body of research has highlighted the detrimental impacts of MPs on diverse marine organisms [8,9,10,11]. However, most experimental studies have concentrated on the deleterious effects of pristine MPs, a focus that is largely inconsistent with actual natural conditions. In reality, marine organisms are exposed to MPs, which undergo a constant process of physicochemical and biological degradation. The degradation of MPs in seawater is a complex process of physical and chemical changes in their structure and is associated with a complex of factors [12,13]. In the marine environment, human-made polymers are constantly exposed to intense physical (temperature, solar irradiation, wind, and mechanical influences), chemical (atmospheric and dissolved oxygen, dissolved salts, polluting organic and inorganic substances), and biological (microbes, phyto- and zooplankton) influences. It is acknowledged that under controlled laboratory conditions, it is impossible to fully recreate the totality of factors accelerating the degradation of MPs. As a result, in most experimental works, UV irradiation of synthetic polymers is utilized as the most suitable exposure factor, as this mimics the basic natural conditions [14]. As demonstrated by Sarkar et al. [15] and Zha et al. [16], ultraviolet (UV) exposure instigates a series of structural alterations in the polymer chain, including the emergence of additional functional groups, a decline in crystallinity, and an augmentation in hydrophilicity. The deliberate acceleration of plastic degradation is a technique employed by researchers to facilitate the study of the physicochemical characteristics of polymers [12,16,17,18,19]. It is also noted that, as a result of both artificial and natural aging of MPs, including µPS, reactive oxygen species may form on the surface of particles. This, in turn, increases their biological activity [20,21].

The use of artificially aged MPs is relevant for studying the negative effects of such particles on aquatic organisms. To date, experimental work using artificially aged MPs is scarce, yet they demonstrate that such particles have significant effects on various physiological and biochemical processes in organisms [22,23,24]. Moreover, evidence is emerging that degraded MPs have more significant impacts on biota than pristine plastic samples [25,26]. Nevertheless, recent studies on the toxicity of naturally or artificially aged MPs, especially PS, are insufficient to fully assess the effects of this form of plastic on marine organisms.

Sea urchins have traditionally been widely used in ecotoxicological studies as model organisms for testing different types of pollution [27,28,29]. Most of the studies have used gametes and early developmental stages of these marine invertebrates to assess the toxic effects of pollutants, as they have a higher sensitivity to pollution [30,31]. In addition, sea urchins have external reproduction, so gametes are in direct contact with a wide range of pollutants, including MPs present in the environment, during spawning [32,33,34]. It is well documented that the spermatozoon exhibits deficient antioxidant defenses and restricted capacity for DNA damage repair [35,36]. This renders them more vulnerable to genotoxic pollutants than somatic cells and oocytes. The sand dollars *Scaphechinus mirabilis* selected for this study are representative species of sea urchins, widely distributed in the Russian Far Eastern region, and have been the subject of extensive research, with sensitivity to the impact of both natural and anthropogenic factors [37,38,39].

In consideration of the preceding findings, the aim of the study was to investigate the effect of pristine and photoaging primary µPS at three concentrations on the viability and DNA integrity of the sperm of the sand dollars *S. mirabilis*.

## 2. Materials and Methods

### 2.1. Preparation of the Micro-PS Solution

The stock µPS solution with a concentration of 2.5% by mass/volume was purchased from the Baseline Chromtech Research Center (Tianjin, China). Using a Goryaev chamber, the concentration of the microspheres in the stock solution was quantitatively determined by repeating the count three times, resulting in 10^6^ microspheres/mL. The nominal particle size in the stock solution was 0.9 µm with a standard deviation of 0.0264 µm and a distribution coefficient of 0.0120 (characteristics declared by the manufacturer). The solution was stored at 4 °C in accordance with the manufacturer’s specifications. Part of the stock solutions was artificially aged using UV irradiation. The photoaging process consisted of continuously irradiating the PS microspheres using a Supratec HTC 400-241 lamp (Osram, Munich, Germany; nominal power of 460 W, voltage of 230 V, frequency of 50 Hz). The lamp spectral distribution ranges between 275 and 470 nm. The total UV radiation power is 100 W (percent contributed by UV-B is 15%). The distance from the lamp to the surface of the plastic particles was maintained at 3 cm throughout the exposure, which lasted 120 h. According to approximate estimates, this should correspond to 2 weeks of continuous solar irradiation [40]. The polystyrene sample irradiation setup was enclosed in a glass box to prevent accidental contamination. To prepare the working solutions (10, 100, and 1000 microspheres/mL), the stock solution and sterile seawater were used. The choice of concentrations was based on earlier studies of the toxicity of µPS to the sand dollar *S. mirabilis* [38]. No surfactants were present or added to the stock MP suspension. To prevent agglomeration of the µPS, the prepared solutions were subjected to 30 min of ultrasonic treatment in a Sapphire ultrasonic bath before the start of the experiment.

### 2.2. Description of the Experiments

In the course of the experiment, adult specimens of the sand dollar *S. mirabilis* were collected in the Alekseev Bay of Popov Island (Peter the Great Bay, Sea of Japan) from a depth of 4–4.5 m. The adult specimens were transported to the laboratory of the Popov Island Marine Experimental Station, which is part of the Pacific Oceanological Institute (POI FEB RAS) within 30 min in dry thermo-containers (8–10 °C). Subsequently, the specimens were acclimatized for a period of 48 h to a temperature range of 18–19 °C. Following this acclimatization period, gametes *S. mirabilis* were acquired by inducing spawning through the injection of a 0.5 M KCl solution. Eggs were handled following standard procedures [41]. We collected sperm from each male into 10 mL of sterile seawater and then diluted the concentrate 1:9 with seawater. Control tests showed fertilization success was over 95% for all pairs. The working sperm-to-egg ratio was maintained at 200:1.

To assess the toxicity of the pristine and photo-irradiated PS, spermatozoa were incubated in the test solutions (pristine µPS at concentrations of 10, 100, and 1000 microspheres/mL, and UV-irradiated µPS at concentrations of 10, 100, and 1000 microspheres/mL) for a duration of 1 h at a temperature of 18 °C. The control group was incubated under the same conditions in sterile seawater for 1 h. After incubation, spermatozoa from the control and experimental groups were used to assess cytotoxicity (resazurin test), DNA damage (comet assay), and fertilization efficiency.

### 2.3. Fertilization Assay

This protocol was adhered to in accordance with the guidelines set out in EPS 1/RM/27 [42]. Subsequently, fertilization was conducted in pure sterile seawater, and the proportion of zygotes was quantified after 20 min. The formation of a fertilization membrane was used as the visual criterion for successful fertilization. Counting was performed on four parental pairs, each of which was replicated three times (n = 12), with each replicate containing at least 100 zygotes.

### 2.4. Fourier-Transform Infrared Spectroscopy

Fourier-transform infrared (FTIR) spectra were acquired using an IRAffinity-1S instrument (Shimadzu, Kyoto, Japan) configured for attenuated total reflectance (ATR). The measurement parameters included a wavenumber range of 4000–400 cm^−1^, 32 scans per spectrum, and a spectral resolution of 4 cm^−1^. 10 measurements were performed for each experimental group. The background signal was acquired under identical conditions relative to air. Subsequent spectrum processing was carried out with the LabSolutions IR 2.27 software (Shimadzu, Kyoto, Japan). We normalized the spectra to the reference peak at 1452 cm^−1^ as an initial processing step. The content of the functional groups in all µPS samples was determined based on the carbonyl index (CI) [43] and the hydroxyl index (HI) [20] by calculating the ratio of the peak areas in the ranges shown in Table 1.

### 2.5. Resazurin Cytotoxicity Assay

The analysis procedure is based on the method described by Czekanska [44], with minor modifications. The principle of the analysis is that inside the cell, resazurin, a weakly fluorescent indicator dye, undergoes enzymatic reduction in the mitochondria to a bright fluorescent pink resorufin dye. The processed dye is released from the healthy cells into the medium, resulting in a visible color change from blue to pink, the intensity of which reflects the number of viable cells. A 500 µL aliquot of incubated *S. mirabilis* sperm was sampled for each replicate. To this, 50 µL of a 10-fold resazurin solution in phosphate-buffered saline (PBS, pH 7.4) was added, and the mixture was incubated for 1 h at 19 °C on a TS-100C thermoshaker (Biosan, Riga, Latvia). Following incubation, colorimetric analysis was performed using a UV-2550 spectrophotometer (Shimadzu, Kyoto, Japan) by measuring absorbance at 570 nm and 600 nm.

### 2.6. Comet Assay

The level of DNA damage in *S. mirabilis* sperm was assessed using an alkaline comet assay according to a previously established protocol [40]. Briefly, sperm were immobilized in 1% low-melting-point agarose and applied to slides. The cells were then lysed for one hour to remove membranes and proteins. Subsequently, the slides were subjected to alkaline unwinding for 40 min, followed by electrophoresis in an alkaline buffer at 2 V/cm for 20 min. Finally, the slides were neutralized with 0.4 M Tris-HCl (pH 7.4), fixed in ethanol, and stained with SYBR Green I.

Visualization was performed with a fluorescence microscope (Carl Zeiss, Oberkochen, Germany), and comet analysis was conducted using CASP software version 1.2.2 (CASPlab, Wroclaw, Poland). A total of 600 comets were enumerated from four parental pairs, with 50 comets scored per biological replicate.

In addition, to show the differences in the effects of high concentrations of aged and pristine plastic in more detail and clearly, comets were divided into separate groups according to the degree of DNA damage, based on the work of Mitchelmore et al. [45], with the author’s modifications. Comets with a 3% difference in DNA damage were separated into individual groups.

### 2.7. Statistical Analysis

The results of the experiment were processed using the MS Excel and Statistica 10 software packages. The data did not achieve normality (Levene’s and Shapiro–Wilk’s tests), and therefore a non-parametric Kruskal–Wallis ANOVA was performed, followed by a series of Mann–Whitney tests to identify significant differences between pairs of data sets. A *p*-value of less than 0.05 was considered statistically significant.

## 3. Results

### 3.1. Changing the Structure of Polystyrene

**Figure 1:**
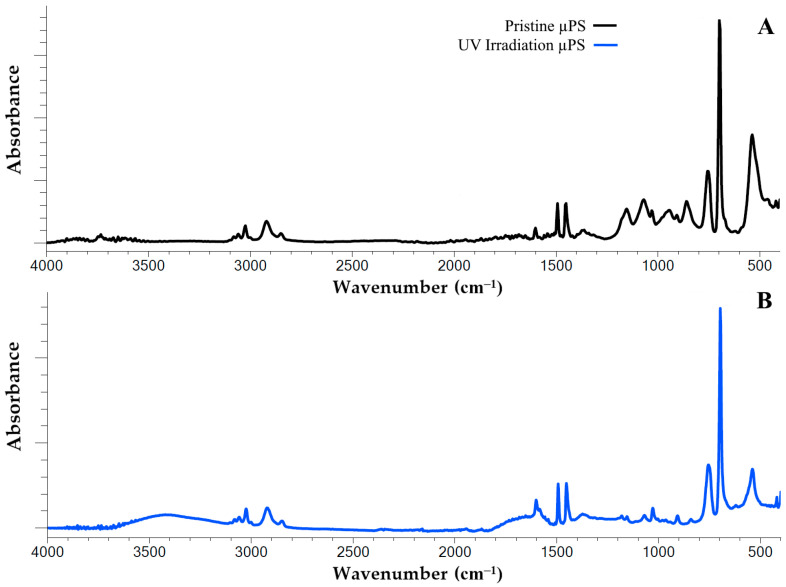
FTIR spectra of pristine (**A**) and artificial aging by 120 h with UV irradiation (**B**) polystyrene microspheres.

We used infrared spectroscopy (FTIR) to detect the structural changes in the polystyrene polymer chain resulting from continuous UV irradiation. Using this method, we recorded significant changes in the IR spectrum of artificially aged µPS samples relative to the spectrum of the pristine particles (Figure 1).

The IR spectrum of the polystyrene exhibits characteristic peaks that are associated with the vibration of the aromatic C=C bonds at the following wavelengths: 1600 cm^−1^, 1492 cm^−1^ and 1452 cm^−1^. In addition, the infrared spectrum of PS exhibits a peak at 3026 cm^−1^, which is attributed to the vibration of the valence aromatic C–H bonds in the benzene ring. In the pristine samples utilized in this study, two peaks (698 cm^−1^ and 756 cm^−1^) were identified. These peaks are attributed to out-of-plane C–H bond vibrations, suggesting the presence of substituents within the benzene ring.

A comprehensive analysis of the IR spectrum of the artificially aged samples revealed substantial alterations in their structural composition. It was thus determined that following continuous UV irradiation for a period of 120 h, a generalized peak within the range of 3100–3600 cm^−1^ was formed in the structure of the polystyrene polymer chain. This finding indicates the appearance of new functional groups, specifically hydroxyl groups (O–H bonds) (Figure 1A). Additionally, an increase in the peak intensity within the range of 1600 to 1750 cm^−1^ was observed, which is associated with the formation of functional carbonyl groups (Figure 1B).

The alterations observed in the infrared spectra of the samples under investigation are indicative of the accumulation of diverse oxidative degradation by the products of PS during artificial aging. In order to more clearly visualize the degree of UV-induced oxidative degradation of the polystyrene polymer chains, the corresponding degradation indices, i.e., the carbonyl index (CI) and the hydroxyl index (HI), were used (Table 1). It is evident from the indices presented that UV irradiation has a significant impact on the levels of carbonyl and hydroxyl groups in the sample. The data indicates a three-fold increase in the carbonyls content and an almost ten-fold increase in the hydroxyls content in response to UV irradiation.

### 3.2. Changing in Sperm Viability

**Figure 2:**
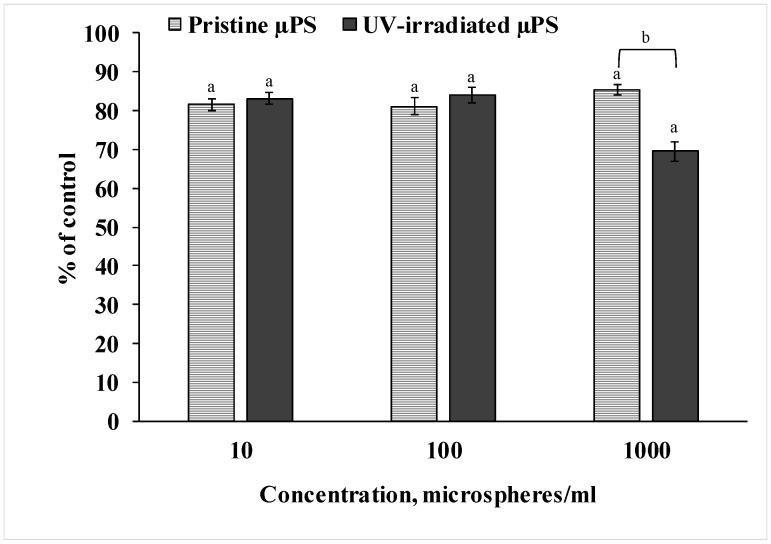
Results of resazurin test after µPS exposure (mean ± sd, n = 12), a—difference from control, b—difference between pristine and UV-irradiated µPS (*p* < 0.05).

To measure the viability of the sperm of *S. mirabilis* sand dollars exposed to both pristine and photoaged primary µPS, we used the resazurin test, as shown in Figure 2. Analysis of the data showed that spermatozoa exposed to pristine µPS at all concentrations tested had reduced ability to reduce resazurin to resorufin by 15–20% relative to the control values. In spermatozoa exposed to UV-irradiated µPS, this decreased by almost 20% at concentrations of 10 and 100 particles/mL and by 30% at a concentration of 1000 particles/mL. The results indicate a decrease in viability based on the metabolic activity of sperm from both experimental groups. Also, the result of the experiment indicates that artificially aged µPS had a greater effect compared to fresh particles at a concentration of 1000 particles/mL.

### 3.3. Destruction of Sperm DNA

**Figure 3:**
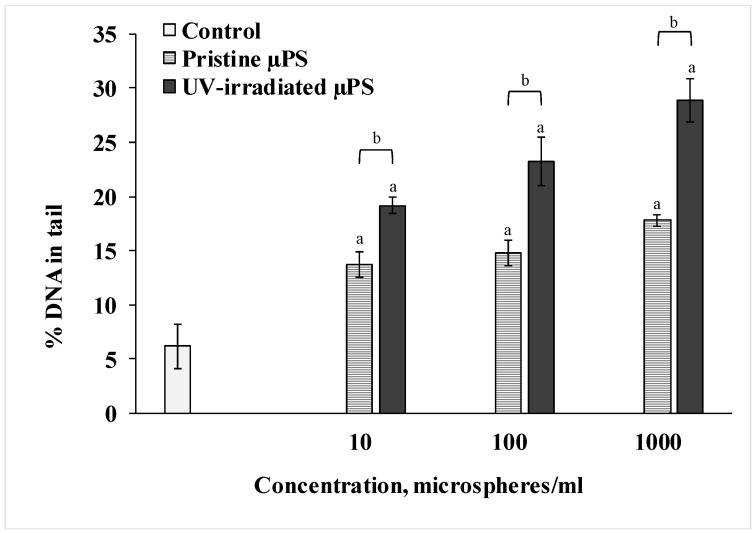
Comet assay results (mean ± sd, n = 600). a—difference from control, b—difference between pristine and UV-irradiated µPS (*p* < 0.05).

We used the DNA-comet method to determine the degree of destructive changes in the spermatozoa of the control and experimental groups. The results are presented in Figure 3. According to the data of the Comet assay, we can see that short-term exposure to pristine µPS resulted in a significant dose-dependent increase in the fragmentation of the nuclear DNA of the spermatozoa of *S. mirabilis*. Thus, in the control group of sperm, the average percentage of DNA in the tail was 6.2 ± 2.03%. When exposed to pristine µPS in concentrations from 10 to 1000 particles/mL, this parameter increased to 13.75 ± 1.2%, 14.75 ± 1.18% and 17.8 ± 0.51%, respectively. In addition, DNA damage was more pronounced when exposed to aged µPS compared with pristine microgranules. The highest percentage of DNA damage (28.91 ± 2.21%) was detected in sperm exposed for 1 h to water containing 1000 particles/mL photoaged µPS.

**Figure 4:**
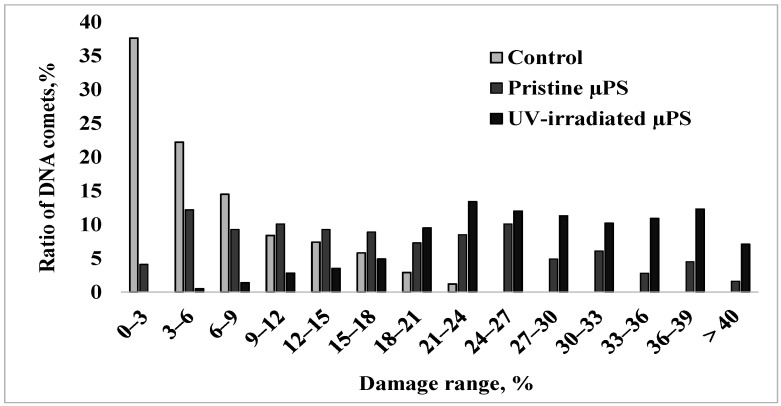
The distribution of *S. mirabilis* sperm of control and experimental groups (1000 microspheres/mL) according to DNA damage.

For a more detailed comparative analysis of data on the nature of nuclear DNA destruction of spermatozoa of control and experimental groups with an exposure concentration of 1000 microspheres/mL, the formed comets were categorized into groups with a genome damage interval of 3% (Figure 4).

In the control group, the degree of damage to the largest number of cells (approximately 90%) did not exceed 15%, and the maximum DNA content in the comet tail was 18–24% and was observed in only 4% of cells. Upon short-term exposure to pristine µPS, about one-third of the sperm formed comets with 21 to 33% damage, and cells with a high degree of DNA damage exceeding 35% were present. Exposure to aged µPS resulted in a significant increase in the number of cells with maximal nuclear DNA fragmentation.

### 3.4. Fertilization Assay

It is worth noting the fact that the evaluation of the effect of the studied µPS on the fertilizing ability of spermatozoa did not reveal any changes. Notably, sperm fertilization efficiency remained high (up to 97%) and was independent of the level of DNA damage.

## 4. Discussion

The present study details a short-term experiment that simulates the interaction between primary plastic MPs and male gametes of the sand dollars *S. mirabilis* under natural conditions. However, it should be noted that the studies were conducted under controlled laboratory conditions in order to minimize extraneous influences typical of the marine environment. In addition, in this experiment, we used concentrations of MPs that exceed those currently observed under natural conditions. Such an approach (using significantly high concentrations of the pollutant) is imperative to study the vulnerability of cellular structures and to determine the mechanisms of toxicity, which is also applicable to this work. Thus, laboratory studies on the short-term effects of plastic on aquatic organisms use concentrations of 10 to 10,000,000 particles/L [46,47,48].

As sea urchins are characterized by external reproduction, the release of their gametes during the spawning season occurs directly into the surrounding seawater, where fertilization and further embryo development take place. Sperm are part of the plankton and are thus unable to avoid water masses contaminated with various chemicals, including but not limited to MPs [33,34]. At this particular juncture, spermatozoa are found to be the least protected, with their outer membranes and receptors being directly exposed to pollutants [30,31,32].

Concurrently, the number of studies that have confirmed the fact that both natural and artificial degradation of plastics leads to an increase in their biological activity is increasing. It was established that aged MPs of varying polymers exhibited heightened toxicity in comparison to fresh samples, exerting an influence on the physiological and biochemical processes of diverse organisms. For instance, exposure to aged PS particles with diameters of 3 and 10 μm resulted in elevated mortality rates of stage II larvae of the acorn barnacle *Amphibalanus amphitrite*, in addition to the delayed development and survival of nauplii of these barnacles [49]. Furthermore, photoaging under simulated sunlight PS MPs induced histological changes and diminished the gonadosomatic index in the Pacific oyster *Crassostrea gigas* [26]. In another study, UV-irradiated particles obtained by grinding disposable plastic cups at concentrations of 100–1000 μg/L increased malondialdehyde and 8-hydroxy-2′-deoxyguanosine levels and lipofuscin accumulation in nematodes *Caenorhabditis elegans* [13]. Sampsonidis et al. compared the toxicity of UV-irradiated and non-irradiated polyethylene particles (PE-MPs) in feeding freshwater fish *Perca fluviatilis*. The authors showed that all particles caused DNA damage and nuclear abnormalities in the liver and muscle tissue of the fish. Fish fed aged PE-MPs caused more acute overall effects, initiated stronger stress responses, inflammation and cellular damage in the tissues studied [50]. Also, a 30-day period of feeding *Zophobas morio* larvae with weathered polystyrene samples decreased the total antioxidant capacity. A significant increase in superoxide dismutase activity and malondialdehyde content and glutathione-s-transferase level in the tissues of the experimental group of larvae was detected [51]. A similar pattern was observed here: the artificial aging of µPS particles amplified their biological activity. This was evidenced by a marked increase in their cyto- and genotoxic effects on *S. mirabilis* sperm.

To ascertain the negative impact of the µPS under scrutiny, a highly sensitive cytochemical test was applied. This test characterizes general stress in cells and is utilized as an indicator of the viability of hydrobiont cells [52,53]. The resazurin test showed a decrease in the intensity of resazurin recovery in the sperm of both experimental groups relative to control values, pointing to a significant downregulation of cellular metabolism. This effect was more pronounced in spermatozoa exposed to UV-irradiated µPS at a concentration of 10^6^ particles/L. Furthermore, the Comet assay was utilized in this study to evaluate DNA damage in individual cells. This method is also widely used in ecotoxicological studies and is a sensitive tool for determining the genotoxic properties of various pollutants [54,55]. The data obtained from the study demonstrated that the spermatozoa from the control group exhibited a minimal level of DNA damage, estimated at 6%. This DNA fragmentation may represent a survival mechanism, where the accumulation of single- and double-stranded breaks and alkali-labile sites sustains essential cellular processes [56]. Short-term exposure of *S. mirabilis* spermatozoa to µPS resulted in increased levels of DNA damage, with UV-irradiated particles causing genome disruption to a greater extent than pristine particles.

To date, there have been few studies investigating the genotoxic properties of MPs in marine invertebrates. The works based on the Comet assay method focused on the effect of synthetic polymers on the hemocytes of bivalves. DNA strand breaks were detected in hemocytes of the mollusk *Scrobicularia plana*, which were exposed to PS at a concentration of 1 mg/L (20 μm) for two weeks [57]. Similar DNA damage to hemocytes of the mussel *Mytilus galloprovincialis* was observed after their uptake of PE-MPs [53]. In another experiment, chronic exposure to PE-MPs resulted in a rapid increase in DNA degradation of mussel hemocytes [58]. There are also works demonstrating the genotoxicity of PS-MPs similar in size to the particles used in our experiment. Exposure of μPS (1 μm in diameter) for 24 h to the shrimp *Neocaridina davidi* and the branchiopod *Daphnia magna* resulted in significant DNA damage and a significant increase in the comet tail length of individual cells relative to the control groups. Another study investigated the genotoxicity of 1 µm PS particles on the branchiopod *Ceriodaphnia dubia*. The authors demonstrated that these particles induce DNA damage in individual cells, reaching up to 28% at a concentration of 8500 µg/L [59].

The primary and distinctive feature of the results obtained throughout the course of our experiment was the study of the process of genome integrity destruction in germ cells. It is imperative to acknowledge the significance of spermatozoa in the context of genome formation, given their role as the primary agents responsible for the transfer of paternal DNA. The integrity of their DNA is of paramount importance, as it serves as a fundamental component in the development of the subsequent generation. This underscores the crucial need to preserve the integrity of spermatozoa, as they are equally crucial to the process of genome formation as oocytes. However, male sea urchin gametes contain highly condensed DNA, lack advanced antioxidant defenses and are incapable of repairing significant DNA damage [35,60]. In the case of successful fertilization of eggs, unrepaired sperm DNA damage is transmitted to zygotes and contributes to the genome of the next generation [36]. The introduced significant DNA damage can then affect the success of early embryonic development, up to its complete arrest and embryonic death. It is believed that the increase in sperm DNA damage under the influence of various pollutants is responsible for their embryotoxicity [60,61].

However, the experiments conducted did not reveal a decline in the fertilization capacity of spermatozoa upon DNA damage. This suggests that both fresh and aged µPS particles induce biochemical shifts that are sufficient to damage the genome but not sufficient to reduce fertilization success. In a previous study, we proposed that there is a threshold for DNA damage in *S. mirabilis* sperm and that fertilization success decreases when this threshold is exceeded [9]. This finding is consistent with observations in other sea urchin species, where fertilization success also appears to be uncorrelated with the level of sperm DNA damage [62]. Moreover, analogous outcomes were attained in the experimental studies with other genotoxic agents. Thus, it was determined that the short-term exposure of mussel and oyster spermatozoa to benz[a]pyrene and diuron caused dose-dependent damage to their genome, with no effect on fertilization success [60,63]. Furthermore, Devaux et al. [36] and Santos et al. [64] observed that fish sperm with a high level of DNA damage retained their ability to fertilize after exposure to methyl methanesulfonate and diuron.

The precise mechanism by which MPs exert their genotoxic effect remains to be fully elucidated. It is challenging to comprehend the initiation of genome destruction processes in the context of MPs, including polystyrene, due to their chemical inertness and inability to penetrate cellular structures. It is hypothesized that the genotoxic effect of such particles is associated with the ability to increase the generation of reactive oxygen species (ROS) and oxidative stress, exceeding the capabilities of the antioxidant defense system [65]. Utilizing model experiments, the researchers detected the activation of the antioxidant system in various marine organisms upon exposure to MPs, including the Pacific oyster larvae *Crassostrea gigas* [66], the rotifers *Brachionus koreanus* [67], and the branchiopod crustaceans *Daphnia magna* [68]. It is hypothesized that ROS are the primary agent responsible for the damage to DNA molecules [53,57]. In the context of spermatozoa, it has been hypothesized that MPs, characterized by their hydrophobic nature, have the capacity to adsorb onto the outer membranes of cells. This process has been suggested to result in the disorganization of the receptor-signaling system, which, in turn, has been proposed to precipitate the occurrence of oxidative stress [69,70].

Furthermore, the toxicity of MPs is attributable to the presence of a wide range of chemical compounds in the structure of the synthetic polymers. These can be low molecular weight fragments of mono- and oligomers, stabilizers, catalysts, dyes, plasticizers, and so forth. It has been hypothesized that these compounds may possess genotoxic properties [71,72].

To confirm the reasons for the higher toxicity of artificially aged µPS and to explain the effects of exposure to such particles on the spermatozoa of the sand dollars, we characterized the changes in the structure of PS that were induced by UV exposure. To achieve this objective, the technique of infrared spectroscopy (FTIR) was utilized, a method that has been identified as one of the most prevalent instruments in the detection of chemical alterations in the surface structure of artificial polymers [20,73,74]. As demonstrated in Figure 1, UV exposure resulted in the formation of carbonyl and hydroxyl functional groups within the surface structure of the polystyrene particles. It is evident that the increase in the peaks observed in the range of 1635–1765 cm^−1^ is indicative of the formation of ketone, carbonyl and aldehyde products [20,75]. Concurrently, the enhancement of spectral lines within the 3420–2550 cm^−1^ range is characteristic of hydroxyl bond vibrations [41]. In general, the formation of these oxygen-containing functional groups may indirectly indicate the development of oxidative degradation processes of polystyrene polymer chains, which are characteristic of both artificially aged (induced by UV irradiation) and aging in a natural marine environment [76,77,78,79].

The photo-oxidation of PS has been found to result in multiple polymer chain breaks and produce a wide range of chemical products that can leach into the environment [15,19,75]. Furthermore, alterations in the chemical structure of polystyrene affect its physical and mechanical properties. As demonstrated in the extant literature, the exposure of synthetic polymers to UV radiation results in the formation of pores and microcracks on the polymer’s surface. In addition, there is an increase in the crystallinity and hydrophilicity and a decrease in the particle size due to fragmentation [10,61,80,81]. It is hypothesized that the biological activity of MPs is enhanced by oxidative degradation, which is caused by artificial or natural aging. Moreover, today it is considered that free radical reactions make a significant contribution to the destruction of polymers [19,21,41,82], the direct and indirect consequences of which are effects on biological systems. A substantial body of research suggests that MPs’ toxicity stems from their capacity to generate reactive oxygen species (ROS) within biological systems, thereby triggering oxidative stress [11,83,84]. However, the mechanisms of ROS generation remain unclear and largely hypothetical. One prevailing theory posits that the relatively hydrophobic nature of synthetic polymers allows MPs to disrupt cell membrane receptor-signaling systems, which may subsequently activate pro-oxidative pathways [69,85]. This approach also makes it possible to explain the toxic processes recorded in the presented article and other works devoted to the effects of aged MPs [10,21,77,86].

## 5. Conclusions

In the present study, the toxic effects of pristine and UV-aged polystyrene µPS at three concentrations were investigated during short-term exposure to gametes of the sand dollars *S. mirabilis*. Infrared spectroscopy results showed significant structural changes in the polystyrene, thereby confirming the degradation of the polymer upon UV exposure. That artificially aged µPS exhibited a more pronounced effect compared to pristine particles, as evidenced by a decline in sperm viability and an escalation in DNA degradation. Despite employing concentrations of MPs that exceed those observed in natural environments, the findings obtained from this study appear to be particularly pertinent considering the escalating pollution of the marine ecosystem by MPs. The results obtained in this study serve to both corroborate and expand upon the previously stated hypothesis concerning the toxicity of aged plastic fragments, thereby underscoring the necessity for further study into the toxicity of aged MPs on marine invertebrates. Future studies should focus on their interaction with the gametes of marine organisms at the biochemical level, using ecologically relevant concentrations and longer exposure times. Taken together, such data will help predict the development of the long-term adverse effects of MPs pollution.

## Figures and Tables

**Table 1:** Quantification of functional groups in pristine and photoaging polystyrene microspheres.

Index	Formula (Wavelength, cm^−1^)	Pristine µPS	UV Irradiation µPS
CI [43]	(1635–1650)/1452	0.42 ± 0.05	1.12 ± 0.07 *
HI [20]	(3420–3550)/1452	0.03 ± 0.01	0.35 ± 0.04 *

*—Values differed significantly from the control (*p* < 0.05).

## Data Availability

Data is contained within the article.
